# Role of matrix metaloproteases in idiopathic pulmonary fibrosis

**DOI:** 10.1186/1755-1536-5-S1-S9

**Published:** 2012-06-06

**Authors:** Annie Pardo, Moisés Selman

**Affiliations:** 1 Facultad de Ciencias, Universidad Nacional Autónoma de México, Ciudad Universitaria, CP 04510, México DF, México; 2 Instituto Nacional de Enfermedades Respiratorias "Ismael Cosío Villegas", Tlalpan 4502, CP 14080, México DF, México

## Abstract

Lung fibrosis is the final common pathway of a large variety of chronic lung disorders, named interstitial lung diseases. The most aggressive form is the idiopathic pulmonary fibrosis [IPF] characterized by alveolar epithelial cell injury/activation, expansion of the fibroblast/myofibroblast population, and the exaggerated accumulation of extracellular matrix [ECM] components which ultimately result in the destruction of the lung parenchyma. Several matrix metalloproteases [MMPs] are upregulated in the IPF lungs and have been shown to actively participate in the pathogenesis of the disease through extracellular matrix remodeling and basement membrane disruption. However, MMPs can also breakdown molecules that mediate cell-cell and cell-ECM interactions, and can activate growth factors and growth factor receptors indicating that they likely contribute to other local biopathological processes such as apoptosis, migration, proliferation and angiogenesis.

## Introduction

### Idiopathic pulmonary fibrosis

Lung fibrosis is the final result of a large and heterogeneous group of lung disorders, known as interstitial lung diseases (ILD) [1]. One of the most common and unquestionably the most aggressive ILD is idiopathic pulmonary fibrosis (IPF) with its histopathologic pattern of usual interstitial pneumonia (UIP) [1,2]. IPF is a chronic, progressive, irreversible and usually lethal lung disease of unknown etiology, with a median survival of 2-3 years from diagnosis in affected patients.

Most patients are between 50-70 years old and the frequency of the disease increases markedly with age. However, the mechanisms linking aging with the disease remain elusive. Its histological hallmark, UIP, is characterized by a variegated appearance [temporal heterogeneity] resulting from irregular juxtaposition of fibrotic scarring, honeycomb change, some interstitial inflammation and normal lung [3].

IPF seems to have a long asymptomatic period, where alveolar epithelial cell microinjuries and activation are occurring in different small areas of the both lungs, primarily in the basal and subpleural regions. After a time, the anatomical and functional magnitude of the lesions reach a threshold, and the symptoms [primarily dyspnea] become visible.

Most evidence indicates that epithelial cells are critical in the initiation and progression of the disease. Thus, aberrantly activated alveolar/bronchiolar cells produce the cytokines and growth factors responsible for the migration and proliferation of local fibroblasts [e.g. platelet derived growth factor (PDGF)], as well as of circulating fibrocytes (e.g. CXCL12) and its transition to myofibroblasts, usually through the secretion of transforming growth factor beta-1 (TGFβ1) [1,4-6]. In addition, αvβ6 an integrin which is able to activate latent TGF-β and that is expressed at low or undetectable levels in normal lungs is strongly upregulated in IPF within epithelial cells lining the alveolar ducts and alveoli [7].

Also, epithelial cells contribute to the expansion of the fibroblast population through the epithelial to mesenchymal transition (EMT) as it has been demonstrated with three different approaches in IPF lungs [8-10]. Turning an epithelial cell into a mesenchymal cell requires a profound change in genetic and epigenetic programs leading to alterations in morphology, cellular architecture, adhesion, and migration capacity [11]. Interestingly, emerging evidence indicates that tumor-associated matrix metalloproteases (MMPs) can stimulate processes associated with EMT. Thus, it has been shown that the exposure of mouse mammary epithelial cells to MMP-3 stimulates epithelial-mesenchymal transition [12]. Likewise, other MMPs, such as MMP-2 and MMP-9 mediated E-cadherin disruption as a key step in epithelial tubular cell EMT indicating that may be also implicated in the fibrotic response [13]. Moreover, recent data indicate that MMP-9 has a higher capacity than that of Snail in eliciting the development of EMT [14].

Independently of their origin, fibroblasts and myofibroblasts accumulate as small clusters (foci) in subepithelial areas arranged in a linear fashion within a pale staining matrix. Overlying epithelium consists of hyperplastic pneumocytes or columnar non-ciliated bronchiolar cells [3]. Finally, these mesenchymal cells secrete exaggerated amounts of extracellular matrix molecules, primarily fibrilar collagens that provoke an extensive structural disorganization in the lung microenvironment where alveolar-capillary units are lost and replaced by scarring and cysts [honeycombing]. Importantly, many of the processes that result in this severe architectural remodeling involve an uncoordinated regulation and expression of several matrix metalloproteases.

### Matrix metalloproteases

MMPs are the M10 family of endopeptidases that belong to the MA zinc-containing clan of metallopeptidases and to the metzincin subclan of proteases [15,16]. In humans the MMPs family consists of 23 members that are codified in 24 genes including duplicated MMP-23 genes [17]. Most of the MMPs are secreted enzymes, although there are some membrane types MMPs (MT-MMPs). Importantly however, some of the secreted MMPs have been found inside the cell and probably acting on intracellular substrates [18,19].

According to their structural and functional characteristics, MMPs family members have been classified into six subgroups that include: collagenases, gelatinases, stromelysins, matrilysins, membrane-type MMPs (MT-MMPs), and other MMPs [20]. Based on structure MMPs have been also classified as archetypal MMPs, matrilysins, gelatinases, and furin activable MMPs [21].

MMPs are tightly regulated at the transcriptional and post-transcriptional levels and their expression, usually low or undetectable under physiological conditions, increased under some processes such as wound healing [22]. After secretion, MMPs are also regulated by activation of the precursor zymogens and by inhibition through endogenous inhibitors, tissue inhibitors of metalloproteases (TIMPs). Uncontrolled MMP activity results in tissue damage and functional alterations.

The contribution of MMPs to extracellular matrix remodeling is complex. They are not only responsible of matrix degradation but also may contribute processing a variety of bioactive mediators such as growth factors, cytokines, chemokines, and cell-surface-receptors modulating their activity either by direct cleavage, or releasing them from extracellular matrix bound stores.

### MMP-1 and MMP-7 are strongly upregulated in idiopathic pulmonary fibrosis

**Figure 1:**
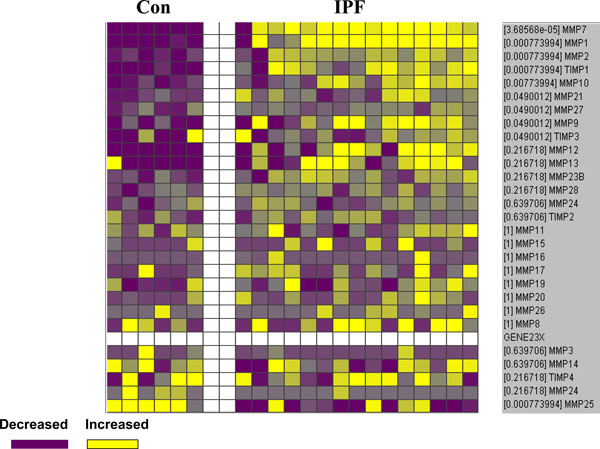
**MMPs and TIMPs expression levels by microarray analysis from controls and IPF lungs**. Increased genes are shown in progressively brighter shades of yellow, and decreased genes are shown in progressively darker shades of blue. Genes shown in gray are not different between the groups.

The transcriptional signature and immunohistochemical analyses of IPF lungs have revealed that several MMPs, primarily MMP1 and MMP7 are among the molecules that are more significantly overexpressed compared with control lungs [16,23] (Figure 1).

#### MMP1 is increased despite the progressive accumulation of fibrillar collagens

MMP1, also known as collagenase-1, is the archetype of MMPs capable of degrading types I and III fibrillar collagens. Therefore, its over-expression in IPF lungs, where interstitial collagens are progressively depositing is an unsolved paradox. Furthermore, excessive MMP-1 is involved in diseases that, in contrast to fibrosis, are characterized by exaggerated extracellular matrix degradation, such as rheumatoid arthritis and lung emphysema [24,25]. A possible explanation for this paradox was revealed by the observation that in IPF lungs the enzyme is expressed primarily by epithelial cells whereas is virtually absent in the fibroblastic foci where the interstitial collagens are being secreted [26,27].

The role of the epithelial expression of MMP1 in IPF is presently unknown, although it might contribute to the formation of the cystic spaces (honeycombing), characteristic of this disorder. The strong epithelial expression of MMP1 in IPF lungs might be also implicated in cell migration as occurs in skin wound healing [28].

Given the prominent upregulation of this enzyme in IPF lungs, we recently tested the frequency of the 2G/2G genotype at -1,607, which is associated with increased gene expression, in a cohort of IPF patients. We found that this genotype is increased in the patients with this disease, but additionally, by sequencing the MMP-1 promoter we revealed a putative gene-environment interaction between the T/G SNP at position -755 and smoking in this disease [29]. This is an important finding because several studies performed in sporadic or familial cases of IPF have shown that smoking is considered a strong risk factor of IPF [6].

#### MMP7 is one of the genes most consistently increased in idiopathic pulmonary fibrosis

Several studies indicate that dysregulation of MMP-7 is associated with IPF [23,30,31]. Similar to MMP-1, the increased immunoreactive protein is also found primarily expressed by the abnormal alveolar epithelium [23]. However, the excessive production of MMP-7 has been clearly associated with the tissue fibrotic response since the MMP7 null mouse is protected from bleomycin-induced lung fibrosis [23]. The profibrotic role of MMP7 might be multiple considering its broad substrate specificity that includes basement membrane and extracellular matrix components. Additionally, this enzyme processes numerous bioactive substrates including FAS ligand, β4 integrin, E-cadherin, pro-HB-epidermal growth factor, plasminogen, pro-TNF-α, syndecan, and insulin growth factor binding protein-3 (IGFBP-3), and diverse studies support a role for this enzyme in apoptosis, inflammation and innate immunity.

Interestingly, in the alveolar epithelial cells of IPF lungs MMP7 colocalize with osteopontin, and application of weakest link statistical model to microarray data indicate a significant interaction between both molecules suggesting that this interaction may have an important effect on the IPF development [30]. This hypothesis is supported by the findings that MMP7 is induced and activated by osteopontin while the later is cleaved and activated by MMP7 [32].

Interestingly, the analysis of renal tissue gene expression associated with aging MMP-7 has been found among the genes significantly up-regulated in very old kidneys that showed higher rates of histopathological changes including glomerulosclerosis and interstitial fibrosis [33]. This is an important finding since as mentioned IPF is considered an aging-related lung disease [6].

### MMP-1 and MMP-7 may serve as diagnostic biomarkers

Regardless their effects in the architectural remodeling in IPF lungs, MMP-1 and MMP-7 may play a role as biomarkers for the differential diagnosis. Thus, in a recent study it was found that both enzymes are significantly higher in the serum of patients with IPF compared to patients with hypersensitivity pneumonitis, sarcoidosis and chronic obstructive pulmonary disease [34]. A similar trend in gene expression of MMP7 and MMP1 is found in the lungs of patients with IPF and HP, supporting the notion that the changes in peripheral blood concentrations are reflective of the lung gene environment and constitute a disease-specific signal. Moreover, levels of circulating MMP-7 appeared to predict outcome since MMP7 higher concentrations of this enzyme negatively correlated with percent predicted forced vital capacity and percent predicted carbon monoxide diffusing capacity.

### MMP- 3 may contribute to epithelial-mesenchymal transition

Recently, it was reported enhanced expression of MMP-3 in IPF, compared with control lungs and MMP-3-null mice were protected from bleomycin-induced pulmonary fibrosis [35]. Interestingly, in vitro treatment of lung epithelial cells with MMP-3 resulted in activation of the β-catenin signaling pathway, via cleavage of E-cadherin, with the subsequent induction of epithelial-mesenchymal transition. The authors propose that MMP3 plays a role in the pathogenesis of IPF, through the induction of epithelial-mesenchymal transition.

### MMPs may participate in the traffic of fibrocytes to the IPF lungs

It is known that fibrocytes, bone-marrow derived progenitor cells, are a source of fibroblasts/myofibroblasts that participate in the mechanisms of wound healing and fibrosis in many tissues. Recently, we demonstrated the presence of fibrocytes in the lungs from patients with IPF, and provided evidence for their principal mechanism of chemotaxis, the CCL12/CXCR4 axis [5]. Lately, we found that fibrocytes strongly express several MMPs including MMP-2, MMP-9, MMP-8 and MMP-7 [36]. To elucidate the role of MMP-8, MMP-2 and MMP-9 in fibrocytes biology we tested the hypothesis that these enzymes may participate in the process of tissue migration. In this context, we found that the migration of fibrocytes through collagen I is highly associated with the expression of collagenase MMP-8 while its migration through basement membrane-like proteins was associated to MMP-2 and MMP-9 since both transmigration assays were blocked by specific inhibitors. Therefore, synthesis of these enzymes may play an important role in transendothelial and tissue migration of fibrocytes and also contribute in the remodeling of ECM during the development of IPF.

### MMP-9 is associated with the lack of thy-1 receptor in lung fibroblasts

MMP-9 is also elevated in IPF, and it is expressed by a variety of cells including alveolar epithelial cells, neutrophils and fibroblasts in fibroblastic foci [27]. The finding that lung fibroblasts in IPF are synthesizing MMP-9 is intriguing since these cells do not express this enzyme in vitro, but a recent finding in our laboratory open some light to this result. It is well known that fibroblasts differ in a variety of phenotypic features, including the expression of Thy-1 a glycophosphatidylinositol-linked glycoprotein. In this context, it was shown that fibroblasts in IPF lungs are Thy-1 negative, whereas most fibroblasts from normal lungs are Thy-1 positive [37]. Recently, we demonstrated that Thy-1 (-) lung fibroblasts stimulated with TGF-β1 expressed MMP-9 while Thy-1 (+) cells did not [38]. Moreover, treatment of Thy-1 (-) fibroblasts with β-glycan, which sequester TGF-β, thereby functioning as a receptor antagonist, abolished MMP-9 induction. TGF-β1-induced MMP-9 in Thy-1 (-) fibroblasts depended on the activation of ERK1/2 signaling pathway [38]. Therefore, it can be suggested that in the microenvironment of IPF lungs, the mesenchymal cells from the fibroblasts/myofibroblasts foci, which do not express Thy-1, stimulated by the epithelial-produced TGF-β1 synthesize MMP-9. Importantly, MMP-9 is able to activate TGF-β contributing to enhance the pool of active TGF-β [39]. Taken together, these findings indicate that TGF-β induction of MMP-9 in Thy-1 (-) fibroblasts could be part of a fibrogenic positive feedback loop.

## Conclusion

Idiopathic pulmonary fibrosis is a devastating disease characterized by epithelial cell activation and the expansion of the fibroblasts/myofibroblasts population in the alveolar septa and alveolar spaces which is followed by aberrant deposit of extracellular matrix. The pathogenic mechanisms remain elusive, but a growing body of evidence supports that several matrix metalloproteinases are implicated in the abnormal remodeling of the extracellular matrix including basement membranes, although an antifibrotic effect of some of them under certain circumstances can not be ruled-out. In addition, the biological consequences of the MMPs-cleavage of chemokines, cytokines, and growth factors in the lung microenvironment are presently unknown. A better understanding of the molecular mechanisms that are involved in the pathogenesis of the disease as well as those that regulate the local balance between accumulation of extracellular matrix and their degradation will be critical to set the MMPs in their exact place. Also, an improved understanding of the complexity of MMP proteolysis and their in-vivo substrate repertoires will help to define the fibrosis-specific effects of MMPs in IPF.

## Competing interests

The authors declare that they have no competing interests.
